# Sexually transmitted infections in the era of antiretroviral-based HIV prevention: Priorities for discovery research, implementation science, and community involvement

**DOI:** 10.1371/journal.pmed.1002485

**Published:** 2018-01-10

**Authors:** Jeanne M. Marrazzo, Julia C. Dombrowski, Kenneth H. Mayer

**Affiliations:** 1 Department of Medicine, University of Alabama at Birmingham, Birmingham, Alabama, United States of America; 2 Department of Medicine, University of Washington, Seattle, Washington, United States of America; 3 Department of Medicine, Beth Israel Deaconess Medical Center, Harvard Medical School, Boston, Massachusetts, United States of America; 4 The Fenway Institute, Boston, Massachusetts, United States of America

## Abstract

Jeanne M. Marrazzo and colleagues join PLOS Medicine's Collection on the prevention, diagnosis, and treatment of STIs with a Perspective on HIV research imperatives in our time of effective viral suppression and pre-exposure prophylaxis.

**Summary points**

- Persons living with HIV who achieve sustained viral suppression with antiretroviral therapy can avoid sexual transmission of HIV without using condoms.
- Similarly, pre-exposure prophylaxis with tenofovir-emtricitabine in HIV-uninfected persons is highly effective.
- With this background, rates of sexually transmitted infections are increasing in some HIV-infected populations, and in some at risk for HIV acquisition.
- The implications require reassessment of the alignment and prioritization of HIV research funding, public health policy, and community engagement.

Antiretroviral therapy (ARV) that achieves virologic suppression renders risk of sexual transmission to HIV-uninfected partners insignificant, lending hope that “treatment as prevention” (TasP) can arrest the epidemic [1]. Moreover, persons who appropriately use tenofovir-emtricitabine (TDF-FTC) for pre-exposure prophylaxis (PrEP) can avoid HIV acquisition, and its use is increasing in resource-rich countries [2]. With the gradual uptake of each of these interventions, sexual behaviors have evolved. As ARV enhanced quality of life and, naturally, sexual health, increases in the rates of sexually transmitted infections (STIs) were reported among people living with HIV—notably syphilis, especially among men who have sex with men (MSM) [3]. The high efficacy of TDF-FTC as PrEP has highlighted the fact that individuals living with HIV, and those at risk, can avoid HIV transmission or acquisition in the absence of barrier methods of protection—another blow to arguments in support of routine condom use. As PrEP uptake has gained traction, a “new wave” of increasing STI incidence is gathering strength—and MSM are not the only concern. In sub-Saharan Africa, PrEP is being rolled out in settings where syndromic management is still the standard approach to STI management—clearly, a suboptimal situation. Demonstration projects of PrEP in these settings have not had the capacity or intent to evaluate concomitant shifts in STI incidence at a community level. This will be a critical effort going forward.

The implications of rising STI rates require reassessment of the alignment and prioritization of HIV research funding, public health policy, and community engagement and give rise to numerous questions. Are STIs an inevitable byproduct of biomedical HIV control, and should the answer change our view of sexual health? Do we need to think differently about management of non-HIV STIs (screening, diagnosis, treatment, partner management) in populations at risk for HIV? Is high STI incidence likely to undermine success of TasP or PrEP in the long term or in certain populations? Should new approaches focus on broader spectrum prevention (agents that inhibit HIV and other viruses)? What are the broad implications, including funding and trial design, for clinical STI research?

## What are the data?

Incident chlamydia, gonorrhea, and syphilis have risen sharply among men in the United States and other industrialized countries, with syphilis disproportionately high among MSM. Reports of gonorrhea at the rectal and pharyngeal sites are increasing disproportionately compared to urethral sites, partially due to enhanced screening of extragenital sites. This may be coincident with increased frequency of unprotected anal sex: in San Francisco, increasing proportions of MSM reported condomless anal sex in the preceding 12 months in the National Health Behavior Study from 2005–2014, and persons attending sexually transmitted disease clinics during the years 2007–2013 reported increases in the number of recent male sex partners [4]. Motivation for decreased condom use may include confidence that use of ARV for prevention attenuates transmission risk or the belief that HIV is no longer a serious health concern.

In studies of TDF-FTC PrEP involving MSM, high rates of incident STI have been observed [5,6]. Increasing rates of condomless sex in the context of PrEP may be only part of the explanation for this. STI increases among MSM antedated the PrEP era, including increasing rates among already HIV-infected MSM [7]. Recognition of the impact of treatment on transmission increased over roughly the same time period as PrEP uptake increased and likely also impacted sexual behavior among MSM. By definition, PrEP users are generally individuals with substantial risk for STIs, as well as HIV. Moreover, routine STI testing has been part of these PrEP studies, providing opportunity for enhanced detection of asymptomatic infections.

Are these trends, if widely representative, necessarily bad for STI control? Some modeling suggests that more frequent screening among MSM using PrEP might over time drive down rates of STI, assuming screening increases substantially and STIs are appropriately treated; there is also the possibility that more treatment of gonorrhea might actually promote faster spread of antibiotic resistance [8,9]. Admittedly, evidence from randomized controlled trials would be needed to study these scenarios.

## What is the solution?

There is an urgent need to optimize new advances in ART-based HIV treatment and prevention to reduce incident STI and to explicitly outline the key questions informing the path forward (Table 1). First, the relationship between HIV biology in the anogenital compartments compared to blood is not completely understood. Notably, most studies evaluating HIV genital shedding have identified RNA copies of HIV by nucleic acid amplification, but less is known about replication-competent, intracellular or extracellular transmissible virus. Ultrasensitive assays to detect minute amounts of P24 protein or replication-competent virions may help to clarify this [10]. Can local genital inflammation mediated by soluble immune mediators facilitate breakthrough local replication of the virus despite systemic control that could promote the risk of transmission? Given the success of ART in significantly decreasing sexual transmission of HIV, even in the presence of high STI incidence, it seems likely that breakthrough shedding on suppressive ART will not be a major contributor to the ongoing transmission of HIV.

**Table 1:** Key research questions.

Overall	• Is the high incidence of STI likely to undermine the success of TasP or PrEP in the long term, in certain populations, or with new PrEP agents? • Can approaches focused on broader spectrum prevention (i.e., agents that inhibit HIV and other viruses) be effective for both HIV and STI PrEP? • What are the broad implications, including funding and trial design, for clinical research in STIs and HIV?
Biology and HIV–STI synergy	• When mucosal injury occurs, does the immune environment influence healing time? • What does hormonal contraception do to the interaction of STI and HIV and to the vaginal microbiome? • Are these processes different in the adolescent genital tract? • How does asymptomatic rectal STI and its treatment perturb the rectal mucosal environment and its receptivity to HIV infection? • For non-TDF-FTC PrEP regimens, can inflammation facilitate breakthrough replication that could overcome the effect of PrEP or promote the risk of HIV/STI transmission? • Could HIV cure strategies that involve interventions to “shock” the virus from latent reservoirs release transmissible virus in the genital tract?
Epidemiology of STIs and sexual behavior in the PrEP era	• To what degree is the increased detection of STI in persons on PrEP due to increase screening (ascertainment bias) versus a true increase in acquisition? • How will prolonged PrEP use impact sexual behavior and sexual networks? • How is PrEP utilized in the context of multiple sexual partnerships? • What is the relative contribution of enhanced detection through routine screening among PrEP users and HIV-infected MSM in care versus absolute increases in STI acquisition due to increases in unprotected sex?
Implementation science	• What innovative testing strategies improve STI diagnosis among individuals on PrEP? • What will be the economic and workforce implications of the increase in STI screening we will continue to see with expanding use of PrEP? • Can STI clinics integrate the provision of PrEP as part of their menu of services? • Can primary care settings seeing patients at risk for HIV improve the quality of STI screening and service provision? • What interventions decrease racial/ethnic disparities in PrEP uptake and STIs?
Study design	• How can we leverage HIV prevention studies using the factorial design strategy to “layer on” STI prevention interventions? • What STI prevention strategies are amenable to more efficient studies focused on operational endpoints (i.e., coverage) instead of effectiveness? • Can the stepped wedge cluster randomized trial approach be used more widely to study clinic-based and population-based STI prevention strategies?

**Abbreviations:** PrEP, pre-exposure prophylaxis; STI, sexually transmitted infection; TasP, treatment as prevention; TDF-FTC, tenofovir-emtricitabine

Second, as other PrEP agents emerge, each will need to be evaluated for any “effect” on STI acquisition and transmission. Because TDF/FTC is not active against bacterial pathogens, studies to evaluate use of antibiotics for prophylaxis are being explored. Doxycycline post-exposure prophylaxis among men on PrEP effected decreased incident syphilis and chlamydia, but not gonorrhea [11]. Given the specter of antibiotic-resistant *Neisseria gonorrhoeae*, this approach needs careful evaluation, and neither Public Health England nor the British Association for Sexual Health & HIV have endorsed it for this reason [12].

Third, implementation science needs to be at the forefront of developing, validating, and deploying state-of-the-art efforts to address STIs in real-world settings. Innovative methods of delivering STI testing and treatment to MSM and other key populations at high risk of HIV, especially those on PrEP, and the use of electronic health records and STI surveillance data to focus rapid response efforts aimed at identifying high-yield foci for interventions aimed at STI–HIV control could facilitate optimal resource allocation. Additionally, development of social media apps that enhance sexual health through knowledge dissemination and user interaction can enhance engagement of at-risk populations with appropriate services.

Finally, the design of clinical trials in the current era of PrEP/TasP is evolving and should consider STI-related outcomes as co-primary or secondary endpoints when appropriate. One example is evaluation of possible unintended behavioral change with an open-label PrEP product whose anti-HIV efficacy has already been established. Another is the risk of unintended crossover between study arms when active products become widely available. Interventions might be “layered on” to ensure that biomedical HIV prevention trials offer state-of-the-art prevention for at risk populations.

## Conclusion

In the era of biomedical HIV prevention, STIs are increasing in many populations, offering new challenges and opportunities. PrEP has introduced a tension between HIV and STI prevention that needs to be articulated and confronted: condomless sex is becoming more frequent, and anxiety about HIV acquisition risk can be considerably lessened. That STI incidence is increasing concurrent with the positive, affirming aspects of PrEP and TasP is a reality that should be recognized as an opportunity to promote sexual health, embrace diversity of sexual expression, and develop creative, efficient, comprehensive approaches to the study of STIs for the next decade. This will entail reexamination of the research priorities that span funding organizations, as well as allocation of funding in its current form. Studies primarily aimed at biomedical HIV prevention should incorporate efficient designs to include STI as well as HIV. Finally, it will be critical to engage communities in an active dialogue to advance mutual understanding of how these disease trends are perceived, studied, and managed.

## Abbreviations

ARV: antiretroviral therapy
MSM: men who have sex with men
PrEP: pre-exposure prophylaxis
STI: sexually transmitted infection
TasP: treatment as prevention
TDF-FTC: tenofovir-emtricitabine
